# Development of omics‐based protocols for the microbiological characterization of multi‐strain formulations marketed as probiotics: the case of VSL#3

**DOI:** 10.1111/1751-7915.13476

**Published:** 2019-08-12

**Authors:** Diego Mora, Rossella Filardi, Stefania Arioli, Sjef Boeren, Steven Aalvink, Willem M. de Vos

**Affiliations:** ^1^ Department of Food Environmental and Nutritional Sciences (DeFENS) University of Milan Milan Italy; ^2^ Laboratory of Biochemistry Wageningen University Wageningen The Netherlands; ^3^ Laboratory of Microbiology Wageningen University Wageningen The Netherlands; ^4^ Human Microbiome Research Program Unit, Faculty of Medicine University of Helsinki Helsinki Finland

## Abstract

The growing commercial interest in multi‐strain formulations marketed as probiotics has not been accompanied by an equal increase in the evaluation of quality levels of these biotechnological products. The multi‐strain product VSL#3 was used as a model to setup a microbiological characterization that could be extended to other formulations with high complexity. Shotgun metagenomics by deep Illumina sequencing was applied to DNA isolated from the commercial VSL#3 product to confirm strains identity safety and composition. Single‐cell analysis was used to evaluate the cell viability, and β‐galactosidase and urease activity have been used as marker to monitor the reproducibility of the production process. Similarly, these lots were characterized in detail by a metaproteomics approach for which a robust protein extraction protocol was combined with advanced mass spectrometry. The results identified over 1600 protein groups belonging to all strains present in the VSL#3 formulation. Of interest, only 3.2 % proteins showed significant differences mainly related to small variations in strain abundance. The protocols developed in this study addressed several quality criteria that are relevant for marketed multi‐strain products and these represent the first efforts to define the quality of complex probiotic formulations such as VSL#3.

## Introduction

The global sales of cultures marketed as probiotics is increasing every year, and their application is considered instrumental for improving health and well‐being. However, this growing scientific and commercial interest has not been accompanied by an equal increase in the evaluation of quality levels for the products that are commercialized (Sanders *et al.*, 2014; Kolacêk *et al.*, 2017). In contrast to starter cultures that are under a strict microbiological quality control because their activity is fundamental for the success of the production process of industrial fermentations, probiotics are currently only controlled at the taxonomic level of the strain(s) used and at the number of viable cells (Tumuola *et al.*, 2001; Patrone *et al.*, 2016; Vecchione *et al.*, 2018). Recently, various attempts have been reported that aimed to define probiotic functions. Probiotic core benefits have been described that are shared among commonly used probiotic strains (Hill *et al.*, 2014; Lebeer *et al.,* 2018). Such probiotic benefits comprise operational functions, such as colonization resistance, production of lactic and short‐chain fatty acids, regulation of intestinal transit, normalization of perturbed microbiota, increased turnover of enterocytes or competitive exclusion of pathogens. While our fundamental knowledge of the underlying mechanisms is still limited, an increasing number of these core benefits have been linked to specific molecules, structures or metabolic pathways that can be identified or predicted using molecular approaches.

Based on the above considerations, the question arises how a probiotic product should be microbiologically characterized to establish its quality level. Taxonomic considerations are usually based on sequence analysis of 16S rRNA genes but increasingly include genomic data. Microbial cell viability is currently verified by standard plating procedure, and the data are expressed as CFU per g of product. This is highly relevant since some studies have found that commercial products did not contain the declared viable cell numbers (Ibrahim and Carr, 2006; Lin *et al.*, 2006; Drago *et al.*, 2010; Morovic *et al.*, 2016). However, other aspects should be considered as well as to assess the quality of a probiotic product and this should include the amount and the relative abundance of damaged and dead cells in the product. These data may be considered as an indicator on how the microbial cells have been prepared by the manufacturers. It should be stressed that in some cases dead cells can maintain the probiotic effects exerted by live cells, as reported in studies with cell cultures, animals and human volunteers (Taverniti and Guglielmetti, 2011; De Almada *et al.*, 2016). Nowadays, several techniques based on differential cell staining are available for the quantification of live and dead microorganisms (Kramer *et al.*, 2009; Davis, 2014; Wilkinson, 2018). Moreover, in multi‐strain probiotic products, besides the evaluation of the overall cell viability, also the single species or strain viability should be considered. Several products on the market are blends of different bacterial species, sometimes with the addition of a yeast (Edwards‐Ingram *et al.*, 2007). In these multispecies products, the relative abundance of viable cells of each strain in the blend should be quantified as these could have different shelf life and function. Considering that the evaluation of the viability of different species in a mixture by plate counting could be problematic because of the absence of selective species‐/strain‐specific media, the development of new approaches is needed, such as those based on single‐cell analysis (Chiron *et al.*, 2017). Moreover, genome‐based molecular tools should be applied, such as metagenomics and metaproteomics approaches, to address all DNA or proteins in a sample as to contribute to a complete and exhaustive microbiological characterization.

In this study, we used as a model the multi‐strain probiotic product VSL#3, consisting of eight strains belonging to seven species that have previously been characterized at the genome level (Douillard *et al.*, 2018). We developed a series of culture‐independent, metagenome and metaproteome‐based microbiological characterizations that could be applied for this and other probiotic products to evaluate the species and strain composition, viability and safety, as well as their proteins and some enzymatic activities that could be used to monitor the reproducibility of the biomass production process. While some microbiological characterizations can be routinely applied on all production lots of a commercialized probiotic product (such as the quantification of live and dead cell by single‐cell analysis, enzymatic assays), other characterizations such as shotgun metagenomics, or metaproteomics could be carried out periodically when changes in the biomass production process happened (*e.g.* change in media composition) in order to evaluate the genomic stability of the strain/s and/or the effect of the changes in media composition on the overall proteome. Some of these approaches could be used by producers, customers and regulators to control the quality of probiotic products, as requested recently (Kolacêk *et al.*, 2017; Jackson *et al.*, 2019).

## Results and discussion

### Identity, safety and composition of the VSL#3 product as determined by metagenomics

The genomes of the individual VSL#3 strains have been characterized previously (Douillard *et al.*, 2018). To provide a genomic description of the multi‐strain VSL#3 product at the species and strain level, a commercial sample (lot A) was characterized by deep shotgun metagenomics since this would generate a level of taxonomic depth that cannot be achieved by a standard phylogenetic analysis based on *16S rRNA* gene profiling. Because the relative abundance of each of the eight strains blended in VSL#3 product is not known, a total of 5 Gb data were generated that included over 6 million Illumina HiSeq reads. Using this sequence coverage, a strain with a load of one billion of CFU up to an overall load of 450 billions of CFU (declared in the product label), and therefore representing approximately 0.22% of the blend, should be theoretically covered by approximately 13 333 reads. The results obtained showed that 99.8% of the total reads matched with an identity of > 99% to the genomes of the 8 strains that had been previously described (Douillard *et al.*, 2018). This confirmed the identity of the strains in this commercial VSL#3 product. A minute number of 425 reads (0.0068% of total reads) were found to have no match in the VSL#3_custom_database and were considered as contaminants originating from the production, laboratory or sequencing processes.

**Figure 1:**
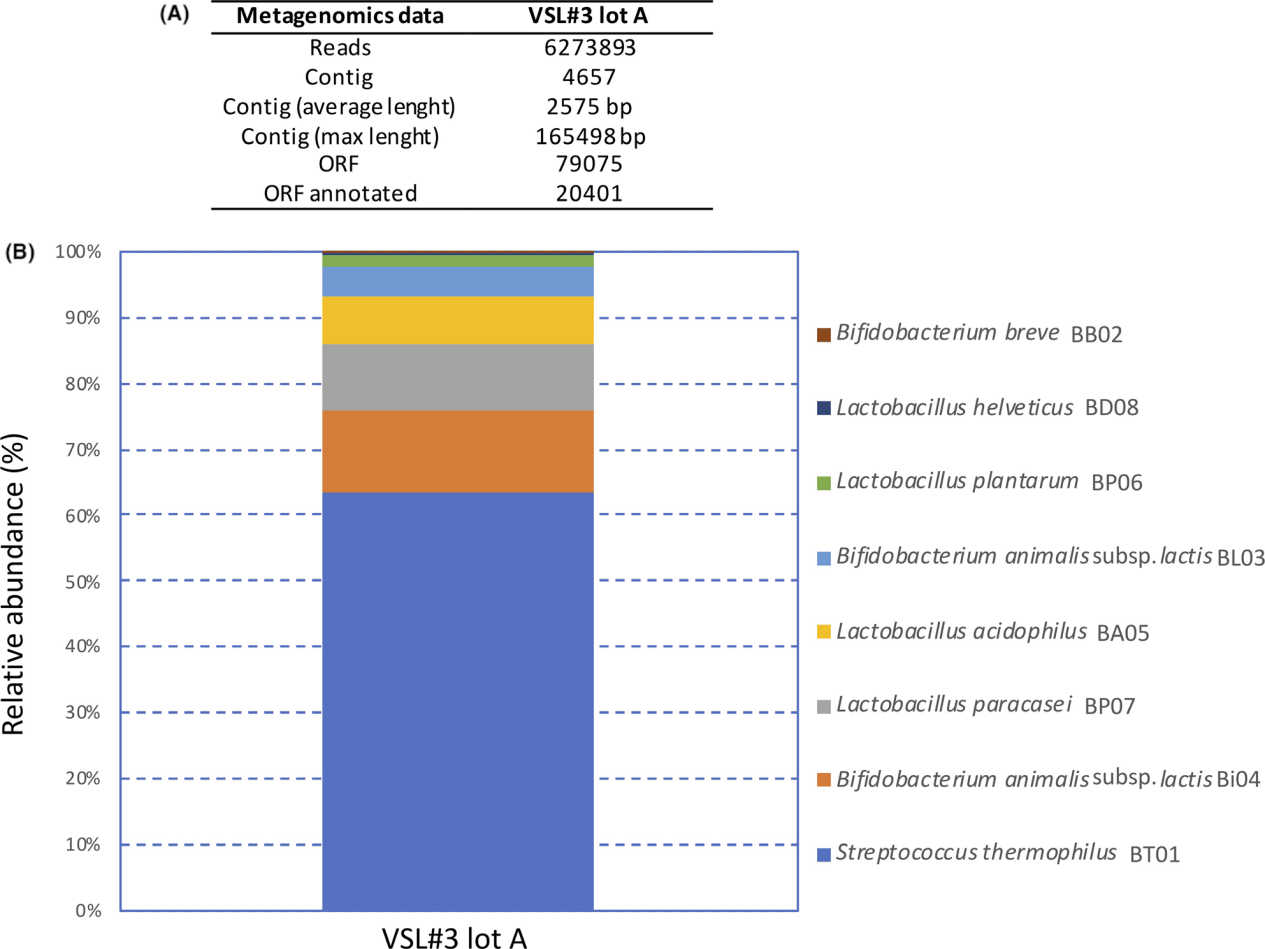
Metagenomics data (A) and relative abundance of VSL#3 strains (B) based on the ORFs count per VSL#3 genome from metagenomic shotgun sequencing of total DNA extracted from VSL#3 lot A assigned by blast against VSL#3_custom_database. The relative abundance of the VSL#3 species was estimated based on the ORFs count per VSL#3 genome. Taxonomy threshold > 95%.

Based on the ORFs count per genome, the relative abundance of the individual VSL#3 strains was estimated (Fig. 1). This does not necessarily reflect the real VSL#3^®^ formulation because the amount of dead cells in the overall cell population could be different among the bacterial strains blended in the product. Nevertheless, the relative abundance estimated from metagenomic data could be used as reference to compare different production lots in terms of homogeneity of species composition. Based on metagenomic data, *S. thermophilus* BT01 was the most abundant (63.4%) species in the blend followed by *B. animalis* subsp. *lactis* (12.7% for strain BI04 and 4.55% for strain BL03; see below), *L. paracasei* BP07 (9.9%), *L. acidophilus* BA05 (7.2%), *L. plantarum* BP06 (1.7%), *L. helveticus* BD08 (0.4%) and *B. breve* BB02 (0.2%). A species‐specific qPCR assay was carried out on total DNA extracted from lot A and lot B, providing a high‐throughput quantification of the VSL#3 species in the blend (Table 1). The qPCR data confirmed the metagenomic analysis with *S. thermophilus* as the most abundant species in the blend followed by *B. animalis* subsp. *lactis* and *L. paracasei*; conversely, *L. helveticus* and *B. breve* showed the lowest count. The qPCR data revealed a significant higher amount of *L. paracasei* in lot B as compared to lot A, whereas the amounts of the other VSL#3 species in the two lots analysed were comparable. Taking into consideration that such multi‐strain product was blended based on viable cells quantified by plate count, the qPCR data, which is biased by the unknown amounts of dead cells, may not reflect the real formulation of the product. The qPCR analysis targeting the *pyk* genes is not able to discriminate between the two highly related *B. animalis* subsp. *lactis* strains (Douillard *et al.*, 2018). However, the metagenomic analysis allowed their identification based on their SNP designation reported previously (Barrangou *et al.,* 2009; Milani *et al.*, 2013), and the SNP abundance was used to estimate their relative abundance (Table 2). Taking into consideration the closed pan‐genome structure of this bacterial species, the superimposable SNP profile between VSL#3 *B. animalis* subsp. *lactis* strains and the references strains Bi07 and Bl04 (Milani *et al.*, 2013) led us to hypothesize an extremely high genomic identity between these strains.

**Table 1:** Quantification of VSL#3 species by qPCR assay.

Species	Lot B	Lot C
*Streptococcus thermophilus*	9.7 × 10^10^ ± 8 × 10^9^	1.6 × 10^11^ ± 1 × 10^10^
*Bifidobacterium animalis* subsp. *lactis*	4.4 × 10^10^ ± 4 × 10^9^	4.3 × 10^10^ ± 5 × 10^9^
*Lactobacillus plantarum*	6.8 × 10^9^ ± 3 × 10^8^	6.5 × 10^9^ ± 4 × 10^8^
*Lactobacillus acidophilus*	4.2 × 10^9^ ± 2 × 10^8^	6.3 × 10^9^ ± 5 × 10^8^
*Lactobacillus paracasei*	4.5 × 10^9^ ± 6 × 10^8^	1.5 × 10^10^ ± 4 × 10^9^
*Lactobacillus helveticus*	1.8 × 10^8^ ± 2 × 10^7^	2.6 × 10^8^ ± 1 × 10^7^
*Bifidobacterium breve*	1.3 × 10^8^ ± 3 × 10^7^	1.7 × 10^8^ ± 2 × 10^7^

**Table 2:** Identification and frequency of *B. animalis* subsp. *lactis* strain‐specific SNPs in the commercial VSL#3 product.

SNP^a^		BI04	Bi07^b^	BL03		Bl04^b^		Number of. reads
6 (A)		Yes	Yes	Yes		Yes		88
8 (G)		No	No	Yes		Yes		17
8 (del)		Yes	Yes	No		No		43
44 (C)		Yes	Yes	No		No		37
44 (del)		No	No	Yes		Yes		12
	BI04						73.6 %	
	BL03						26.4 %	

**a. **SNP number designations are according to Milani *et al.* (2013).

**b.** *B. animalis* subsp. *lactis* reference strains (Milani *et al.*, 2013). Percentage of strain BI04 or BL03 = (number of reads specific for strain BI04 or BL03 x 100)/(number of reads specific for strain BI04 + number of reads specific for strain BL03).

The deep metagenomics data also allowed for a further characterization of *B. animalis* subsp. *lactis* BL03 and BI04. These strains differ in a 54 bp indel within the gene Balac_0771 which codes for a long‐chain acyl‐CoA synthetases involved in cell membrane lipid production. We did not observe this small 54 bp indel in our previous genomic analysis because of variations in the sequence coverage for the two *B. animalis* subsp. *lactis* strains (Douillard *et al.*, 2018). The 54‐bp in‐frame deletion leads to 18 amino acids shorter long‐chain acyl‐CoA synthetases gene in strain BL03 as compared to strain BI04. The 54‐bp deletion was previously associated in *B. animalis* subsp. *lactis* strains to an increased resistance towards oxidative stress (Oberg *et al.*, 2011, 2013). Therefore, we could hypothesize that the VSL#3 *B. animalis* subsp. *lactis* BL03 and BI04 strains might show differences towards oxidative stress, affecting their cell viability in the blend, an aspect that needs to be further investigated.

The metagenomic data were also used to address the safety aspects of the studied VSL#3 product and confirmed what was previously reported by Douillard *et al. *(2018). By using a cut‐off of 80% identity, we detected the *tetW* gene showing identities > 99% with *tetW* of *B. animalis* subsp. *lactis* BL03 and BI04, and *B. breve* BB02, together with the isoleucyl‐tRNA synthetase coding for mupirocin resistance, an intrinsic antibiotic resistance used to selectively cultivate Bifidobacteria (Rada and Koc, 2000; ISO, 29981:^2010^; IDF 220:^2010^). The *tetW* gene detected is present in well‐characterized and commercialized probiotics (Kleerebezem *et al.*, 2003; Garrigues *et al.*, 2010) but has so far not found to be transferable (Gueimonde *et al.*, 2010). The *tetW* gene was previously identified in VSL#3 strains BB02, BL03 and BI04, as was a potential aminoglycoside aminotransferase coding gene in strain BI04 (Douillard *et al.*, 2018). Metagenomic data confirmed the absence of pathogenic islands or genes coding for toxic compounds, thus confirming previous data obtained from the genome analysis of the individual VSL#3 strains (Douillard *et al.*, 2018).

**Figure 2:**
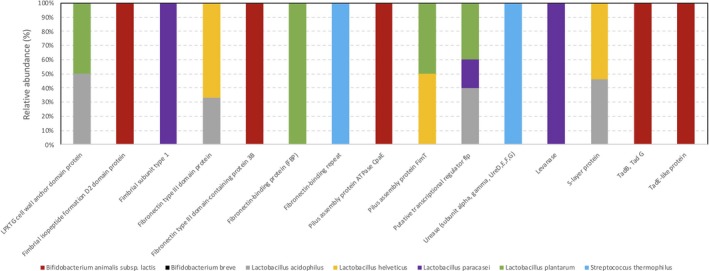
Probiotic functional classification of the reads from metagenomic shotgun sequencing of total DNA extracted from VSL#3 lot A. Data were assigned to general classes with the COG database. Data were expressed as relative abundance with respect to the total reads.

Finally, the shotgun metagenomic data allowed to support a further characterization of the molecular basis of the host interaction of VSL#3 strains. The predicted potential probiotic features coded by VSL#3 metagenome include several cell surface components of *B. animalis* subsp. *lactis*, *L. acidophilus*, *L. helveticus*, *L. paracasei*, *L. plantarum* and *S. thermophilus*, such as pili, fimbriae and fibronectin proteins, and more generally proteins containing the cell‐wall anchoring LPXTG motifs (Fig. 2). While the potential probiotic properties predicted from the VSL#3 metagenome should be further validated for each strain, these cell envelope proteins are known to be involved in adhesion to the intestinal epithelium in well‐characterized probiotic strains, such as *L. rhamnosus* GG and *B. breve* UCC2003 (Kankainen *et al.*, 2009; O’Connell Motherway *et al.*, 2011). Urease‐encoding sequences were only identified in *S. thermophilus,* thus confirming that this enzymatic activity is uniquely present in this species among those constituting the VSL#3 product. A putative *L. paracasei* levanase coding sequence was also identified, thus suggesting the potential ability of VSL#3 *L. paracasei* BP07 to hydrolyse inulin, as recently demonstrated in for an *L. plantarum* strain carrying this conserved gene (Buntin *et al.*, 2017).

### Evaluation of cell viability

**Figure 3:**
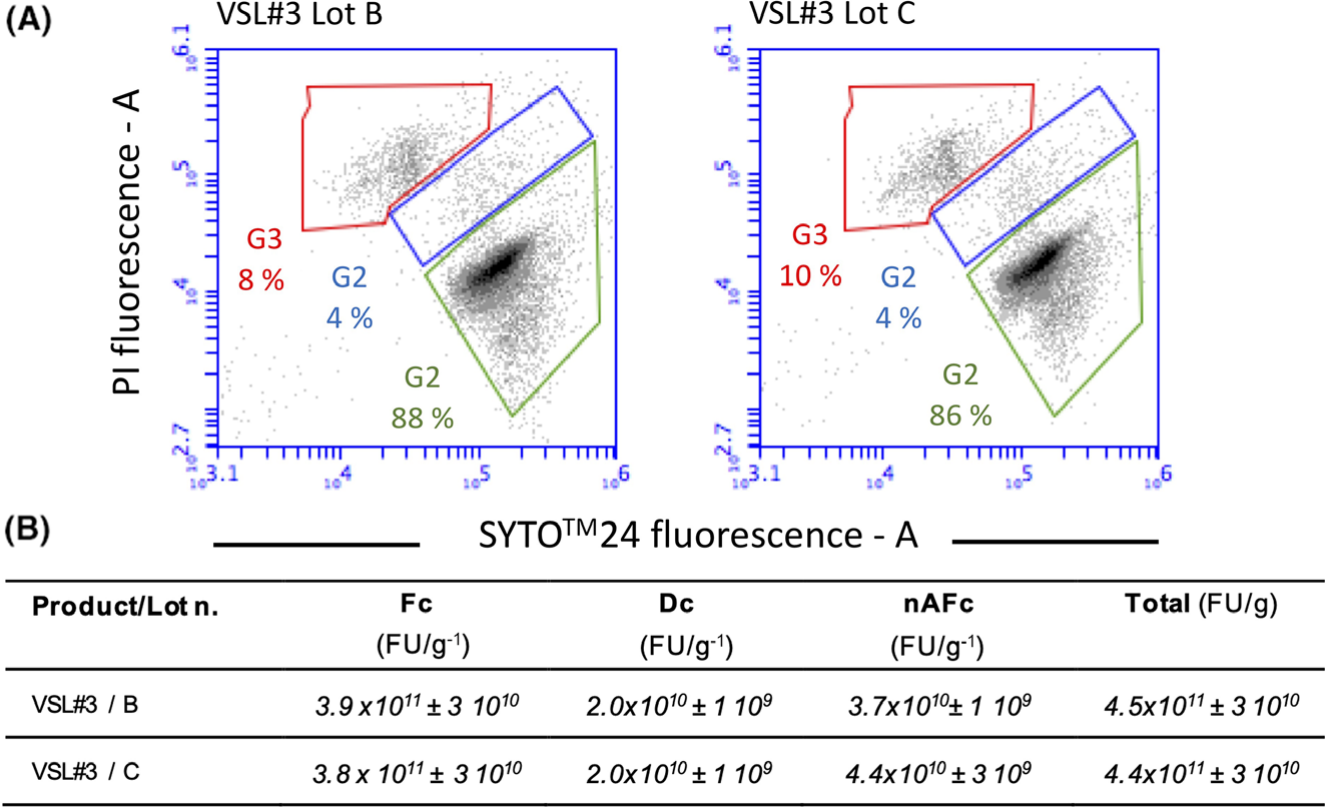
A. Dot‐plots of VSL#3 cell suspensions stained with SYTO®24 and PI. Active fluorescent cells (live cells) (Fc) were identified in the green gate G1, damaged cells (Dc) were identified in blue gate G2, and non‐active fluorescent cells (dead cells) (nAFc) were identified in the red gate G3. Lot B and lot C are indicated in the figure. B. Quantification of Fc, Dc and nAFc. Statistically significant differences were determined by an unpaired Student's *t* test (*P* < 0.05). No significant differences were detected for Fc (*P:* 0.7040) and Dc (*P:* 1.0000). Significant differences were detected for Dc (*P*: 0.0186).

According to the accepted probiotic definition (Hill *et al.*, 2014), the number of live bacterial cells in a probiotic product represents a fundamental requirement. Nevertheless, the overall quality of the probiotic product should also take into consideration the amount of damaged and dead cells already present in the marketed product. The publication of the ISO 19344 IDF 232 (2015) specified a standardized method for the quantification of active and/or total lactic acid bacteria and probiotic strains in starter cultures used in dairy products by means of flow cytometry. We therefore applied an optimized flow cytometric protocol to evaluate live, damaged and dead cells in two commercial lots B and C of the multi‐strain VSL#3 product. The two VSL#3 lots showed a homogeneous distribution of live, damaged and dead cells (respectively, gated in G1, G2 and G3) independently of the expiration date of each lot number (Fig. 3). Live cells accounted for approximately 3.8‐3.9 10^11^ CFU g^−1^, well above the amount of live cell declared in the label (1.0 10^11^ CFU g^−1^). Dead cells were between 8 and 10% of the overall population in both lots, thus highlighting a good optimization of the biomass production process. It is worth to mention that while cells gated in G3 are considered dead, the fate of cells gated in G2, that is those cells showing a moderate cell membrane damage and therefore showing both SYTO24 and PI fluorescence, is not known. It is known that some of the viability of such damaged cells may be rescued (Amor *et al.*, 2002). However, they also could completely lose their viability and therefore they were not included in the count of viable cells.

### In situ analysis of enzymatic activities: β‐galactosidase and urease

Probiotics are living cells that can rapidly respond and adapt to changing conditions in their environment. Therefore, numerous factors including culture preparation and preservation can affect probiotic cell activities (the core benefits) and probably influence the specific host–microbe interactions required for probiotic effects in the digestive tract (Marco and Tachon, 2013; Sanders *et al.*, 2014). Hence, we have developed protocols for the measurement of two enzymatic activities, β‐galactosidase and urease activity, which could be linked to changes in the biomass production process parameters and media composition. The selection of β‐galactosidase activity was based on the fact that 7 up to 8 VSL#3 strains contain a β‐galactosidase‐encoding gene in their genomes. It is known that in *S. thermophilus*, β‐galactosidase gene expression and activity is strictly linked to the energetic status of the cells and the carbon source used in the medium formulation, and it is therefore a good candidate to monitor the reproducibility of the production process (Van d Bogaard *et al.*, 2000). As expected, a considerable amount of β‐galactosidase activity was observed (Table 3). The two lots analysed showed activity values ranging between 8.8 and 9.3 AU, irrespective of the expiration date of the analysed lot, thus indicating a high stability of this enzymatic activity in the bacterial blend, which was also confirmed by the high cell viability.

**Table 3:** β‐galactosidase activity of three VSL#3 lots.

Sample	Expiration date	β‐galactosidase activity (AU)
Lot B	06/2018	8.8 ± 0.3
Lot C	06/2019	9.3 ± 0.3

AU, (mOD_420nm_ min^−1^), statistically significant differences were determined by an unpaired Student's *t* test (*P* < 0.05). No significant differences were detected, *P*: 0.1108.

**Figure 4:**
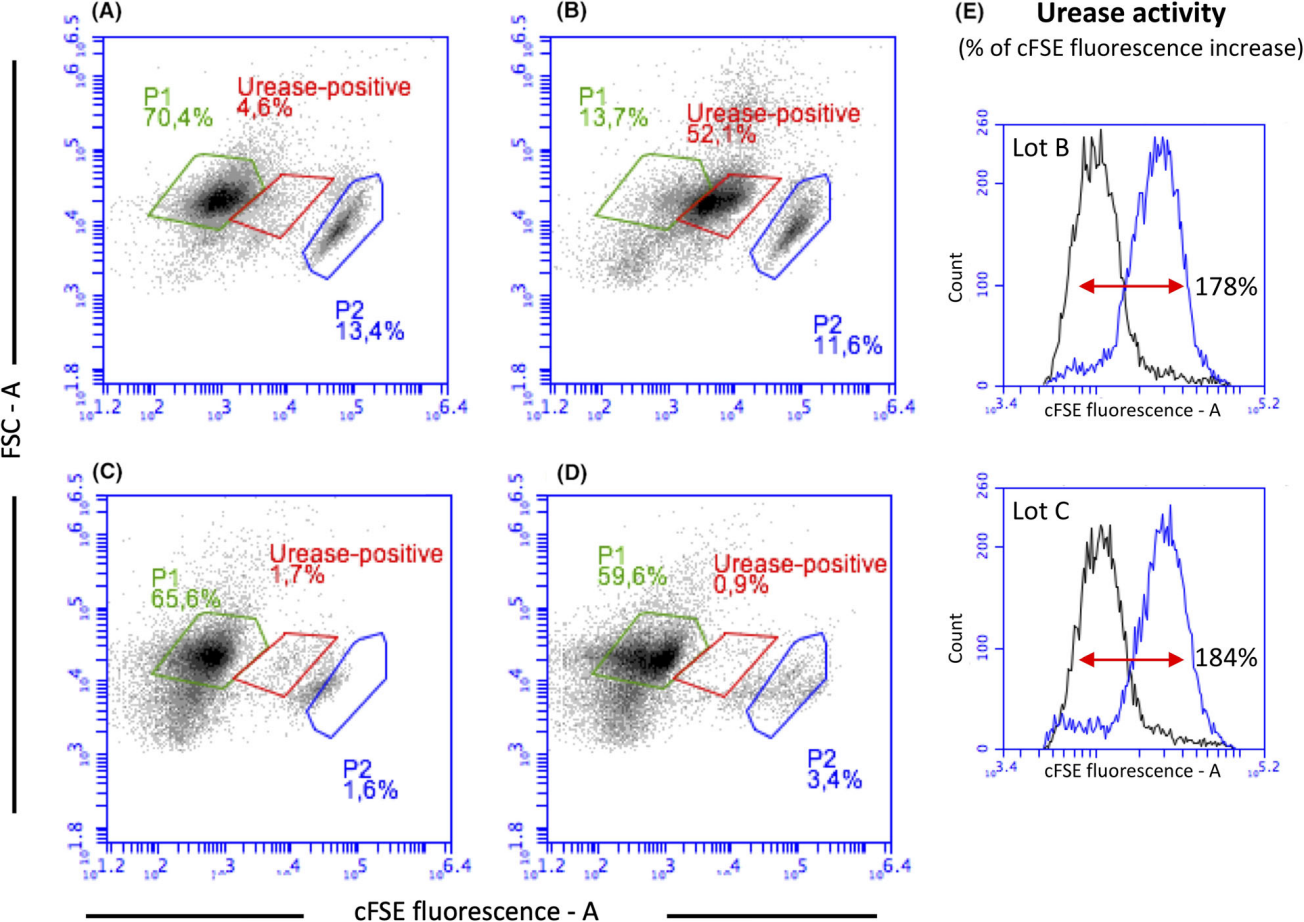
Dot‐plots of VSL#3 cell suspensions stained with cFSE, before (A) and after the addition of 10 mM urea without (B) and with pretreatment of cells with the membrane uncoupling CCCP (C), and with pretreatment of cells with the urease inhibitor flurofamide (D). (E) Histogram showing the urease‐dependent shift in cFSE fluorescence in VSL#3 lots B and C. The analytical imprecision of urease activity analysis based on cFSE fluorescence shift was < 20 %.

Although urease activity has been associated with several pathogens, the human gut microbiota urease was more recently considered a health‐related factor associated with several bacteria colonizing the human gastrointestinal tract (Mora and Arioli, 2014; Douillard *et al.*, 2018). Urease is supposed to act by modulating the nitrogen availability of gut microbiota and host (Millward *et al.*, 2000). This may be particularly the case under conditions where dietary nitrogen is limiting (Yatsunenko *et al.*, 2012). Moreover, urease activity is present in well‐characterized probiotics strains, such as *B. longum* subsp. *infantis*, *L. reuteri*, *S. salivarius* (Power *et al.*, 2008; Lo Cascio *et al.*, 2010; Wilson *et al.*, 2014), and in the yogurt bacteria *S. thermophilus* (Mora *et al.*, 2004). Since *S. thermophilus* is the dominant species in the multi‐strain probiotic product VSL#3 (see Table 1), and the transcription of urease gene cluster is mainly regulated by pH and nitrogen availability (Mora *et al.*, 2005; Arioli *et al.*, 2007), urease activity was chosen as a second marker to monitor the reproducibility of the VSL#3 production process. Urease activity was measured as a shift of the cFSE fluorescence of a cell suspension exposed to urea (Fig. 4). The cFSE fluorescence is pH‐dependent, and it increases during the alkalization generated by urea hydrolysis and consequent ammonia release. The developed assay allowed to identify the urease‐positive cell population within a blend of urease‐positive and urease‐negative cells, and to evaluate the urease activity as % of cFSE fluorescence increase. The activity results shown here indicate that the urease activity is clearly detectable in both commercial lots of the VSL#3 blend (Fig. 4). In fact, a cytometric population representing 52.1% of the total cFSE‐labelled cell community increased its fluorescence due to urea hydrolysis and a consequent intracellular alkalization (Fig. 4B). As a control, the addition of carbonyl cyanide m‐chlorophenyl hydrazine (CCCP), a membrane uncoupling of the proton gradient which inactivate the urea cell membrane transporter, and flurofamide, a urease inhibitor, inhibited the cFSE fluorescence shift (Fig. 4C and D). The results obtained showed that all lots analysed showed comparable urease activity (Fig. 4E). Since the urease activity was unique for *S. thermophilus* (Douillard *et al.*, 2018) as confirmed by the metagenomic analysis that also showed *S. thermophilus* to have over 50 % abundance, we conclude that *S. thermophilus* in the VSL#3 blend is responsible for the urease activity.

Interestingly, a small part from the main cytometric population gated in P1, a small cytometric population (gate P2 in Fig. 4) showing high level of cFSE fluorescence, appeared to be affected by CCCP and flurofamide treatment. These results clearly indicated that *S. thermophilus* cells were present both in P1 and in P2 cytometric populations. The reasons of the special distribution of *S. thermophilus* cells in the two cytometric populations could be related to a population heterogeneity in terms of chain length or to differences in intracellular esterase activity which is responsible of cFSE activation. Such heterogeneity in a homogenous genetic background is quite common in bacterial populations, and it was previously reported for *S. thermophilus* and in *Lactococcus lactis* among lactic acid bacteria (Arioli *et al.*, 2014; Solopova *et al.*, 2014).

### Metaproteomic characterization of VSL#3: Composition, reproducibility and functionality

**Figure 5:**
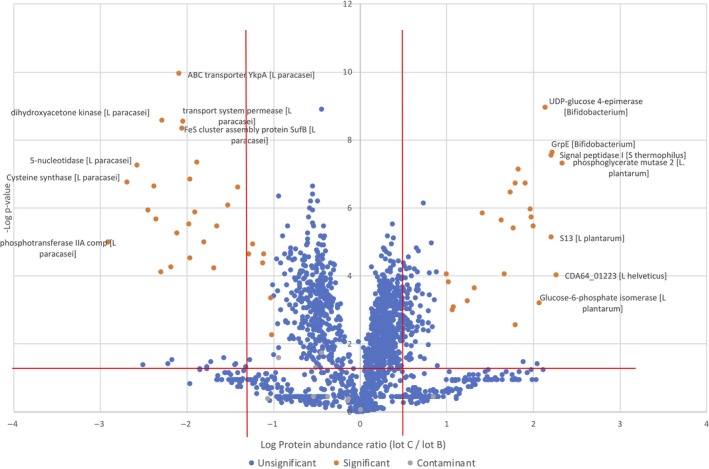
Volcano plot of the protein species (red *P*‐value above 0.05) that differ significantly in abundance between lot B (left) and lot C (right). The significant *P*‐value of 0.1 is indicated by the solid line. Contaminants are indicated by green dots. A listing of the differentially abundant protein species is provided in Table S2.

Metaproteomics approaches have been developed to identify proteins in complex mixtures of microorganisms (Klaassens *et al.*, 2007; Kolmeder and de Vos, 2014). Using advanced bioinformatics, the origins of the proteins can be traced to the production organisms and this has shown to be a useful way to identify the functions of specific microorganisms (Kolmeder *et al.*, 2015). As a consequence, proteomics approaches can be instrumental in characterizing the functionality of probiotic products and notably those derived from multiple species. Moreover, it allows for compositional analysis as well as determining reproducibility and temporal development (Kolmeder *et al.*, 2016). Hence, we applied an earlier developed metaproteomic pipeline to two lots B and C of the multi‐strain product VSL#3, which included triplicate analysis of the extracted proteins by advanced LC‐MS and identification of the proteins by using the genomic information of the VSL#3 product (Douillard *et al.*, 2018). The triplicate analysis showed high protein intensity reproducibility with a Pearson correlation coefficient of on average 0.91. A total of 1659 proteins could be identified by at least 2 peptides in both lots by using these LC‐MS features. Of interest, these proteins could be assigned to all 7 species present in the VSL#3 product (see below). The correlation of the LC‐MS features between the two VSL#3 batches was also high with a Pearson correlation coefficient of on average 0.81, and only 54 proteins (3.2 %) were found to differ significantly between the two lots as visualized in the Volcano plot (Fig. 5). This indicates that both lots have a very high similarity, notably when one takes into account that the multi‐strain products are manufactured by mixing different amounts of freeze‐dried cells of the strains that make up the final formulation. In normal practice, this is realized by adjusting the number of cells based on their viable plate count. However, in this way the formulation contains both damaged and dead cells (see above). The cell quantification carried out by single‐cell analysis highlighted the presence of 3.7‐4.4 x 10^10^ FU g^−1^ of dead cells, the proteins of which contributing to the metaproteome while not being enumerated by plate counting.

### Metabolic interpretation of the metaproteomic analysis

It can be seen from the Volcano plot that 25 proteins are more abundantly present in lot B and 29 more in lot C (Fig. 5). When comparing the proteome data sets of these two commercial lots of VSL#3, two major effects should be distinguished. One is simply that more cells of a single strain have been added, for instance to compensate for a slightly reduced viability, as is commonly observed in industrial manufacturing practice. In this case, the most abundant housekeeping proteins are expected to be more prevalent. The alternative is that cells of a specific strain were harvested a bit later or earlier. This would result in the specific abundance of a set of specific proteins that would respond to different growth and medium conditions, stress or other factors. When analysing the data of the two lots, no clear distinction could be made between these effects as only few differentially present proteins were identified. The possibility that both of these effects could occur simultaneously may also be considered but its analysis would require very deep LC/MS analysis that is beyond the present proof of principle study. Below, the differently abundant proteins in both batches are discussed in some detail.

Apart from a 50S ribosomal protein from *B. animalis* subsp. *lactis*, a fumarate hydratase of *L. plantarum* and a OppA of *L. helveticus*, all other of the 22 proteins that were found to be more abundant in lot B were found to derive from *L. paracasei*. The *L. paracasei* proteins included components of sugar, amino acid and nucleotide metabolism, such as glucoside PTS, oligopeptide and other transport systems, alpha‐galactosidase, dipeptidyl peptidase, and cysteine synthase. Taking into consideration the formulation of VSL#3 product, this may suggest that relative to lot C, slightly more *L. paracasei* cells were included in lot B. This is indeed the case as was concluded from the qPCR‐based quantification of VSL#3 species that showed a higher level of *L. paracasei* in lot B than lot A (see Table 1). A possible explanation could be that this was due to a slightly reduced viability of *L. paracasei* cells in lot B that was compensated by blending in more cells of this species. Ribosomal proteins are usually highly abundant, and hence, it is possible that the presence of *B. animalis* subsp. *lactis* 50S ribosomal protein was indicative of slightly higher amounts of cells of this species were included in lot B as compared to lot C. However, this was not confirmed by the qPCR‐based quantification (Table 1).

When analysing the proteins that were found to be more abundant in lot C than lot B, considerable heterogeneity in the origin of the proteins was observed since they derive from *L. plantarum, L. helveticus, L. acidophilus, S. thermophilus* and *B. animalis* subsp. *lactis.* A slightly higher abundance of *S. thermophilus*, *L. helveticus* and *L. acidophilus* in lot C compared to lot B was also confirmed by the qPCR‐based quantification data (Table 1), whereas qPCR was not able to support differences in cell count between the two lots for *L. plantarum* and *B. animalis* subsp. *lactis*. The distinct proteins from *L. plantarum* included mainly glycolytic enzymes, such as a phosphoglycerate mutase, glucose 6‐phosphate isomerase and fructose‐bisphosphate aldolase, suggesting again that cells of this strain may have been faster growing in lot C than lot B. Only a single protein from *L. helveticus* (hypothetical protein CDA64_01223) was most abundant in lot C and showed high similarity with a cell division or separation protein but has no confirmed function. A similar situation could apply to *L. acidophilus*, from which a single enzyme UDP‐* N*‐acetylmuramoylalanine‐d‐glutamate ligase potentially involved in peptidoglycan biosynthesis was somewhat more abundant in lot C as compared to lot B.

Most of the distinct proteins in lot C derived from *S. thermophilus* and included a signal peptidase, some transporters and components involved in central and energy metabolism. Of interest is the higher abundance in the *S. thermophilus* cells in lot C of a hemin transporter HmeB, which is part of an ABC transporter. While hemin can be incorporated in cytochromes produced by various lactic acid bacteria, this property is not known for dairy strains of *S. thermophilus.* Hence, the abundance of HmeB and other distinct *S. thermophilus* proteins may reflect a general response to the culture medium conditions. The abundant proteins derived from *B. animalis* subsp. *lactis* were predicted to have a variety of metabolic functions and the most abundantly produced proteins included GrpE and GalE. The latter proteins are involved in heat‐shock and other stress response (GrpE) (a heat‐shock and general stress response protein) and GalE (UDP‐glucose 4 epimerase involved in exopolysaccharide (EPS) formation); the specific abundance of these proteins may be interpreted as to indicate that the cells of *B. animalis* subsp. *lactis* in lot C were slightly more stressed than in lot B. According to the supposed differences in susceptibility to oxidative stress between strain BI04 and BL03 due to the allelic variants of the long‐chain acyl‐CoA synthetases gene (Oberg *et al.*, 2011, 2013), it could be also hypothesized that cells of strain BI04 were slightly more abundant in lot B than those of strain BL03.

### Metaproteome‐based functionality analysis of the VSL#3 products

As indicated above, the two investigated lots of VSL#3 are highly similar in composition and it is virtually impossible to attribute differences to different cell numbers or different growth and processing conditions of the species used to produce VSL#3. One way to approach this is to consider housekeeping proteins as a proxy for the cell numbers of the different species, although this would not account for potential differences in cell morphology. A number of such housekeeping proteins have been selected because of their high level of expression, and they can be easily assigned to each VSL#3 species based on their amino acid sequence. They include glycolytic enzymes such as enolase, pyruvate kinase and GAPDH, but also β‐galactosidase and S‐layer proteins due to their high abundance (Table 4). In this context, the proteomic data were consistent with the measured β‐galactosidase (Table 3) that were not significantly different between the two lots, analogously to what reported by the metaproteomic data for *S. thermophilus, B. animalis* subsp. *lactis* and *L. helveticus* β‐galactosidase (Table [Link], [Link], [Link])*.*

**Table 4:** Size‐corrected relative abundance of selected housekeeping proteins as analysed in triplicate in the VSL#3 products lot B and lot C

Description	VSL3 lot B^a^			VSL3 lot C^a^		
	B1	B2	B3	C1	C2	C3
POO10663.1 S‐layer protein [*Lactobacillus acidophilus*]	9.50	9.44	9.52	9.65	9.73	9.69
POO10880.1 Glyceraldehyde‐3‐phosphate dehydrogenase [*Streptococcus thermophilus*]	9.37	9.35	9.31	9.55	9.50	9.59
POO15824.1 Enolase [*Streptococcus thermophilus*]	9.35	9.32	9.39	9.54	9.60	9.56
POO12483.1 Pyruvate kinase [*Streptococcus thermophilus*]	9.20	9.18	9.25	9.34	9.41	9.41
POO18406.1 Glyceraldehyde‐3‐phosphate dehydrogenase [*Lactobacillus paracasei*]	9.40	9.33	9.34	9.23	9.19	9.28
POO19292.1 Pyruvate kinase [*Lactobacillus paracasei*]	9.42	9.45	9.49	9.13	9.23	9.24
POO14182.1 Beta‐galactosidase [*Streptococcus thermophilus*]	8.90	8.84	8.98	9.06	9.00	8.87
POO09985.1 Glyceraldehyde‐3‐phosphate dehydrogenase [*Lactobacillus acidophilus*]	8.88	8.89	8.87	8.99	8.93	8.94
POO11362.1 Pyruvate kinase [Lactobacillus acidophilus]	8.82	8.85	8.85	8.92	9.00	8.97
POO18403.1 Enolase [*Lactobacillus paracasei*]	9.14	9.11	9.13	8.85	8.88	8.97
POO11424.1 Enolase [*Lactobacillus acidophilus*]	8.65	8.62	8.65	8.67	8.72	8.69
POO06052.1 Enolase [*Bifidobacterium animali*s subsp. lactis]	8.26	8.29	8.31	8.63	8.72	8.72
POO05890.1 Glyceraldehyde‐3‐phosphate dehydrogenase [*Bifidobacterium animalis* subsp. lactis]	8.24	8.17	8.28	8.59	8.58	8.46
POO31756.1 S‐layer protein [Lactobacillus helveticus]	8.20	8.26	8.26	8.44	8.48	8.44
POO10183.1 S‐layer protein [*Lactobacillus acidophilus*]	7.78	7.73	7.70	8.30	8.22	8.29
POO10711.1 S‐layer protein [Lactobacillus acidophilus]	7.57	7.67	7.70	8.25	8.22	8.15
POO13015.1 Glyceraldehyde‐3‐phosphate dehydrogenase [*Lactobacillus plantarum*]	7.10	7.32	7.11	8.24	8.19	8.18
POO31797.1 Glyceraldehyde‐3‐phosphate dehydrogenase [*Lactobacillus helveticus*]	7.47	7.51	7.50	7.99	7.88	7.92
POO06020.1 Pyruvate kinase [Bifidobacterium animalis subsp. lactis]	7.54	7.56	7.53	7.75	7.77	7.67
POO14527.1 Pyruvate kinase [*Lactobacillus plantarum*]	6.36	6.42	6.75	7.75	7.80	7.75
POO31994.1 Pyruvate kinase [Lactobacillus helveticus]	7.36	7.35	7.41	7.61	7.70	7.46
POO13018.1 Enolase 1 [*Lactobacillus plantarum*]	6.72	6.62	6.56	7.47	7.54	7.56
POO30743.1 Enolase [Lactobacillus helveticus]	6.69	7.19	7.25	7.24	6.83	7.44
POO08557.1 Glyceraldehyde‐3‐phosphate dehydrogenase [*Bifidobacterium breve*]	6.57	6.43	6.53	6.52	6.37	6.42
POO07455.1 Beta‐galactosidase [*Bifidobacterium animalis* subsp. lactis]	5.70	5.91	5.93	4.30	6.07	6.00
POO30985.1 S‐layer protein [*Lactobacillus helveticus*]	4.30	4.30	4.30	4.30	4.30	4.30
POO31036.1 S‐layer protein [*Lactobacillus helveticus*]	4.30	4.30	4.30	4.30	4.30	4.30
POO31431.1 Beta‐galactosidase small subunit [*Lactobacillus helveticus*]	4.30	4.30	4.30	4.30	4.30	4.30
POO31432.1 Beta‐galactosidase large subunit [*Lactobacillus helveticus*]	4.30	4.30	4.30	4.30	4.30	4.30

**a. **B1, B2 and B3 represent the protein relative abundance of each replicate of lot B; C1, C2 and C3 represent the protein relative abundance of each replicate of lot C.

The S‐layer proteins are highly relevant because these are known to be involved in adhesion to intestinal epithelial cells and extracellular matrix components (Buck *et al.*, 2005). Adhesion is believed to be a requirement for the achievement of certain probiotic effects, such as immunomodulation. More recently, the S‐layer of *L. acidophilus* and *L. helveticus* showed to possess an important immunomodulatory activity. Specifically, SlpA of *L. acidophilus* was found to interact with a major receptor on dendritic cells and regulates dendritic cell immune functions (Konstantinov *et al.*, 2008), whereas SlpA of *L. helveticus* showed anti‐inflammatory effects by reducing the activation of NF‐kB on the intestinal epithelial Caco‐2 cell line and acts as stimulators of the innate immune system by triggering the expression of proinflammatory factors tumour necrosis factor alpha and COX‐2 in the human macrophage cell line U937 via recognition through Toll‐like receptor 2 (Taverniti *et al.*, 2013). Based on the above consideration, S‐layer proteins of *L. acidophilus* and *L. helveticus* should be considered a probiotic core benefit involved in host adhesion and interaction. Hence, the observation that in both lots of the VSL#*3* the S‐layer proteins of *L. acidophilus* and *L. helveticus* are abundant and comprise a similar level testifies for the reproducibility of their probiotic functions. This is of great relevance since the therapeutic dose of VSL#3 is relatively high (450–900 billion cells per day) and the product may be active without further multiplication after consumption.

With the exception of the S‐layer proteins whose expression is known to be high and constitutive (Klotz and Barrangou 2018), the expression of β‐galactosidase and glycolytic enzymes could be affected by changing in the production process parameters and/or media formulation as it was demonstrated for *S. thermophilus* and *Lactococcus lactis* (Van den ogaard *et al.*, 2000; Teusink *et al.*, 2011).

## Conclusions

The multi‐strain VSL#3 product is a high dosage probiotic blend consisting of 8 different strains belonging to 7 *Lactobacillus, Bifidobacterium* and *Streptococcus* spp. This complex product represents an excellent model to develop an appropriate set of omics‐based culture‐independent protocols to evaluate its microbiological quality. The compositional complexity of VSL#3 was first addressed through a metagenomic approach that allowed a detailed taxonomic and functional characterization of the product, giving also information on the relative abundance of the strains constituting the blend and on their safety. The metagenomic results generated here, together with the previous characterization of VSL#3 strains at genomic level (Douillard *et al.*, 2018), provide the basis for controlling the genetic stability of the product along the industrial productions. It is well known that genetic changes due to mutations, repositioning of mobile elements such as plasmids, prophages and insertion sequences/transposons, as well as the immunity‐based CRISPR sequences, may occur in *Lactobacillus, Bifidobacterium* and *Streptococcus* spp*.* (Sanders *et al.*, 2014; Lugli *et al.*, 2019). Two independent lots of VSL#3 were subjected to flow cytometric, biochemical and metaproteomic analysis. The live/dead cell counts confirmed that the analysed lots contained the amounts of live cells declared on the product label. In addition, we measured in the sachets of the two lots the level of β‐galactosidase and urease activity to monitor the reproducibility of the production process. Similarly, the metaproteomic approach showed that both lots contained relatively high levels of the S‐layer proteins of *L. acidophilus* and *L. helveticus,* another set of potential probiotic benefits. Finally, the detailed metaproteomic characterization of the two different lots revealed only a minor number of proteins to be differentially expressed (54 from the 1659 detected), supporting a high standardization level of the fermentation processes applied for the production of the 8 different VSL#3 strains.

There are presently no accepted guidelines to control the quality of the commercialized probiotic products in spite of the demand for these (Kolacêk *et al.*, 2017; Jackson *et al.*, 2019). Previous studies have revealed the potential of omics‐based technology to address the microbial composition at species and strain level in order to verify the correctness of the product label (Morovic *et al.*, 2016; Lugli *et al.*, 2019). Without attempting to set new standards, we here evaluated the potential of high‐throughput and omics‐based approaches to contribute to the required microbiological quality control. We addressed the following microbiological quality criteria that we propose to be useful for future considerations. (i) *Taxonomy*. Correct taxonomy of the strains according to the latest state of the art technology and supported by genomic or metagenomic characterizations. (ii) *Viability.* Several techniques based on differential cell staining are available for the quantification of live and dead microorganisms and should therefore be applied. Single‐cell analysis carried out by flow cytometry is a useful and robust approach. However, it may be difficult to quantify mixed microbial constituents in routine industrial and commercial product quality assessments. Moreover, in multi‐strain products, different species/strains could have a different shelf life in the blend. In this context, flow cytometric methods combining specific antibodies and viability assessment with SYTO^TM^24 (Chiron *et al.*, 2017) together with the FlowFish approach based on fluorescent probes targeted to the 16S rDNA (Rigottier‐Gois *et al.*, 2003) could be suitable approaches. (iii) *Safety*. Probiotic strains should satisfy the safety issues in terms of antibiotic resistances according to EFSA and other recommendations. While these quality criteria could be easily measured in single‐strain product, the MIC evaluation in multi‐strain product could be extremely complex, and metagenomic approaches may have an advantage. (iv) *Reproducibility of biomass production.* Variation in the production process parameters can affect the level of some enzymatic activities associated with one or several strains blended in the probiotic products. Some of these enzymatic activities could be used as a marker to monitor the overall reproducibility of the fermentation processes, and here, we showed the feasibility of using β‐galactosidase and urease for that purpose.

In conclusion, we have shown the feasibility of applying a series of microbiological quality criteria to the multi‐strain product VSL#3. While it is too early to set an industrial standard, we propose that for human intervention trials the used probiotic lots are characterized by these microbiological quality control criteria. This will provide a better comparison between different studies, allow retrospective explanations in case of deviating clinical or health outcomes and contribute to further assessing the role of probiotics in human health.

## Experimental procedure

### The VSL#3 products tested

Different lots of the commercially available VSL#3 product were characterized, and all were obtained from Actial Farmaceutica SRL, Italy. All VSL#3 lots were produced by Nutrilinea Srl and commercialized as sachets containing freeze‐dried bacterial cells to be stored at 4°C. These were shipped to the different laboratories on ice and stored at 4°C as indicated in the product specifications. All tests were performed within the time set by the expiry date.

### Shotgun metagenomic analysis

Ten grams of a commercial sample of VSL#3 (lot A) was suspended to a final volume of 10 ml of TE buffer (1M Tris‐HCl pH 8.0, 0.1M EDTA). 400 µl of a sample dilution corresponding to approximately 10^9^ cells was collected. The cell suspension was subjected to alkaline lysis and DNA extraction according to the protocol described previously (Mora *et al.*, 2004). DNA quantification and quality evaluations were performed with a Qubit fluorometric quantification (Thermo Fisher Scientific, Milan, Italy). The quantified DNA was delivered to BaseClear (Leiden, the Netherlands) for shotgun metagenomic Illumina Nextera XT library preparation and sequencing on Illumina HiSeq 2500 (guaranteed 5Gb HiSeq 2500 paired‐end data per sample). For bioinformatic analysis, paired‐end reads have been merged with FLASH (Fast Length Adjustment of SHort reads) tool (Magoč and Salzberg, 2011) and then assembled into contigs with MEGAHIT tool (Li *et al.*, 2015) using standard parameters. ORF prediction was subsequently performed with Prodigal Gene Prediction Software with –p meta parameter (optimization for metagenomic data). Blast search analysis was performed using BLAST + suite from NCBI. Nucleotide sequences were initially aligned against ‘nt’ database from NCBI consisting of nucleotide sequences from GenBank, EMBL and DDBJ, excluding bulk divisions (gss, sts, pat, est, htg) and wgs entries. A second database (VSL#3_custom_database_nucl) was used to confirm the previous results. VSL#3_custom_database_nucl was built using annotated contigs from the eight bacterial strains present in VSL#3 product (accession number PRJNA388854) (Douillard *et al.*, 2018). Amino acid sequences were initially aligned against ‘nr’ database from NCBI composed by non‐redundant protein sequences from GenPept, SwissProt, PIR, PDF, PDB and NCBI RefSeq (ftp://ftp.ncbi.nlm.nih.gov/blast/db/). A second database (VSL#3_custom_database_prot) was used to confirm the previous results. VSL#3_custom_database_prot was built using annotated proteins from the 8 bacterial strains present in VSL#3 product (accession number PRJNA388854). Both BLASTn and BLASTp analysis were performed with a threshold of 10^‐5^ on the E‐value, and only results that showed a nucleotide identity> 98% and an amino acid identity higher than 90% were used for further analysis. Functional classification was assigned performing a blast analysis against Clusters of Orthologous Groups (COG) database from NCBI. ORFs associated with genes known to code for proteins that could be associated with probiotic effects were detected using keyword searches against previous results from blast against nr and VSL#3_custom_database_prot. A list of proteins and genes of interest was reported previously (Douillard *et al.*, 2018). To identify reads associated with antibiotic resistant genes (ARGs), the data were compared with the CARD (Comprehensive Antibiotic Resistance Database) (McArthur *et al.*, 2013). BLASTp analysis was performed in order to identify the antibiotic resistance‐encoding reads with a threshold of 10^‐5^ on the E‐value. Only sequences with an amino acid identity> 80% were considered. Due to the extremely high level of identity between the two *Bifidobacterium animalis* subsp. *lacti*s present in the mixture (Douillard *et al.*, 2018), differentiation of the two strains has been performed through a SNP analysis based on data reported previously (Milani *et al.*, 2013). The metagenomic sequences were deposited in NCBI’s Sequence Read Archive (SRA) with the following accession number PRJEB31428. The relative abundance of the VSL#3 species was estimated based on the ORFs count per VSL#3 genome. Reads that did not match against VSL#3_custom_database were processed using Geneious Prime software. Reads with length smaller than 80 bp were discarded. MegaBlast analysis was performed against NCBI nt_db (E‐value 10^‐5). Duplicates were removed, and only sequences with grade score > 90% were considered and used for the analysis (grade score is a weighted score for the hit comprised of the E‐value).

### Quantification of VSL#3 species by species‐specific qPCR

Quantification of product species was carried out by qPCR using primer sets targeted to the single‐copy gene *pyk* coding for pyruvate kinase (Table [Link], [Link], [Link]). The qPCR analysis was performed using 5 ng of DNA extracted as above described in a total volume of 15 μl, by using the EvaGreen^TM^ kit (Bio‐Rad Laboratories, Milano, Italy) and following manufacturer’s recommendations. PCRs were performed in triplicate and run on a CFX96 instrument (Bio‐Rad Laboratories). Data were recorded as threshold cycles (*C*_t_), expressed as the mean ± standard deviations, and analysed using Bio‐Rad CFX Manager^TM^ software. A calibration curve for each VSL#3 species that reported the *C*_t_ vs. number of cells was obtained. For this purpose, VSL#3 strains obtained by Actial Farmaceutica Srl were grown as previously described (Douillard *et al.*, 2018). After growth, cells were collected by centrifugation, suspended in BPW buffer (casein peptone 10 g l^−1^, NaCl 5 g l^−1^, Na_2_HPO_4_ 3.5 g l^−1^, KH_2_PO_4_ 1.5 g l^−1^, pH 7.0) and quantified by flow cytometry (FU ml^−1^) as described below. Cells diluted in BPW buffer were subjected to total DNA extraction using DNeasy UltraClean Microbial Kit (Qiagen, Hilden, Germany) according to the manufacturer’s specifications. Five microlitres of DNA was used as a template in qPCR assays with the appropriate species‐specific primer set (calibration curves obtained for each species are shown in Fig. [Link], [Link], [Link]).

### Counting viable, damaged and dead cell by single‐cell analysis

Cell counting was performed on two commercial lots (lots B and C) of the multi‐strain product VSL#3 by flow cytometry using an Accuri C6 flow cytometer (BD Biosciences, Milan, Italy) (Sincock and Robinson, 2001; Gunasekera *et al.*, 2003). A total of 10 g of each lot was suspended in up to 100 ml of BPW buffer and homogenized in a stomacher for 3 min at room temperature. The obtained cell suspensions were analysed by flow cytometer (threshold settings FSC 5000, and 50 μl) without and with labelling with SYTO^TM^24 and propidium iodide (PI) (Thermo Fisher Scientific). All of the parameters were collected as logarithmic signals. The 488 nm laser was used to measure the FSC values. The rate of events in the flow was generally lower than 2,000 events s^−1^. The staining principle is based on a dual nucleic acid staining with cell‐permeant dye SYTO^TM^24, and cell‐impermeant dye PI. SYTO^TM^24 permeates the membrane of total cells and stains the nucleic acids with green fluorescence. PI penetrates only bacteria with damaged membranes, causing a reduction in SYTO^TM^24 fluorescence when both dyes are present. Thus, live bacteria with intact cell membranes fluoresce bright green (defined as active fluorescent cells), bacteria with slightly damaged membranes exhibit both green and red fluorescence (defined as damaged cells), whereas bacteria with broken membranes fluoresce red (defined as non‐active fluorescent cells). The flow cytometric absolute count was performed using the Fluoresbrite^TM^ polychromatic red 2.0 μm microspheres as reference (Polysciences Europe GmbH, Hirschberg an der Bergstrasse, Germany). The SYTO24 fluorescence intensity of stained cells was recovered in the FL1 channel (excitation, 488 nm; emission filter, 530/30), whereas PI fluorescence was recovered in the FL3 (excitation, 488 nm; emission filter, 610/20). Density plots of SYTO24 vs. PI allowed for optimal distinction between the SYTO24‐PI stained cells and instrument noise or sample background. Electronic gates on the SYTO24/PI density plot were used to select and measure the total bacterial concentration (events per ml), active fluorescent cells (AFc), damaged cells (Dc) and non‐active fluorescent cells (nAFc) as described in ISO 19344 IDF 232.

### Flow cytometry‐based measurement of urease activity

Urease activity measurement was carried out in cell suspensions of each lot B and C prepared in as described above but using Mitsuoka buffer instead of BPW (Muto *et al.*, 2010). Cells were stained using the pH‐sensitive fluorescence probe 5 (and 6‐)‐carboxyfluorescein succinimidyl ester (cFSE), based on the method originally described by Breeuwer *et al. *(1996), later improved slightly (Sawatari and Yokota, 2007), and modified as described in Arioli *et al. *(2017). The fluorescence intensity of this probe increases at alkaline pH and decreases at acidic pH. Cell suspensions in Mitsuoka buffer were obtained as described above, diluted to obtain approximately 1000–2000 events μl^−1^ and supplemented with 4 µM cFDASE (Sigma‐Aldrich, Milan, Italy), which is a precursor molecule of cFSE. The suspensions were then incubated for 30 min at 37°C. During this incubation, the membrane‐permeating cFDASE was cleaved by intracellular esterases and the resultant cFSE molecules were conjugated to the aliphatic amines of intracellular proteins. Apart from pH, the intensity of the fluorescence depends on the esterase activity of the cells and could be a species‐ and/or strain‐dependent feature. After the measurement of cFSE fluorescence, the Mitsuoka cell suspension was supplemented with urea 10 mM and incubated at 37°C. cFSE fluorescence was measured every 5‐min interval for 15–30 min. Urease activity was expressed as the % increase of the average cFSE fluorescence of the cytometric population as follows: [(cFSE fluorescence *_after urea addition_* – cFSE fluorescence *_before urea addition_*)/cFSE fluorescence *_before urea addition_*]x100.

### β‐galactosidase activity

Samples of each lot B and C were suspended (1 g in 10 ml) in sterile 50 mM Tris‐HCl buffer (pH 8.0) and quantified by flow cytometry. The cell suspensions (10^8^ CFU ml^−1^) were permeabilized as described by Krishnan *et al*. (2000) with the following modification. First, the cell suspension was supplemented with an equal volume of 10% (v/v in water) toluene. Subsequently, the obtained suspension was incubated for 2 min at 30°C with shaking (100 rpm, Unimax 2010, Heidolph Instruments, Milano, Italy). Finally, the permeabilized cells were recovered by centrifugation (12,000 x g, 5 min), washed twice in Tris‐HCl buffer (pH 8.0) and used immediately for β‐galactosidase assay. Measurement of the β‐galactosidase activity was performed in 200 μl aliquots of permeabilized cell suspension containing 0.2 mg ml^−1^ of 2‐nitrophenyl‐β‐d‐galactopyranoside (Sigma‐Aldrich, Milano, Italy) at 37°C, by monitoring the optical density at 420 nm with a microplate‐reader M680 (Bio‐Rad Laboratories, Hercules, CA, USA) programmed for a reading set of 60 repetitions with intervals of 30 s. The β‐galactosidase activity was expressed in arbitrary units (AU) defined as mO.D._420 nm_ per min as the mean of four independent determinations.

### Metaproteomic analysis

The two different commercial lots B and C of the VSL#3 product were also analysed by metaproteomics. For each analysis, 0.5 g of the product was suspended in 1 ml lysis buffer (4% SDS, 50 mM DTT in 100 mM Tris.HCl, pH 7.6) and transferred to an autoclaved 2.0‐ml screw‐cap tube, containing 0.5 g 0.1 mm zirconia/silica beads and five 2.5 mm glass beads. The suspensions were bead‐beaten at room temperature for three cycles of 60 s with a 5‐s ramp‐up time, at 5500 ms using a Precellys 24 bead‐beating machine. The samples were cooled on ice after every cycle. The suspension was then spun down for 5 min at 21 130 *g*, 4°C, and the supernatant was transferred to a clean 2.0‐ml Eppendorf tube, quantified by Bradford analysis, and stored at −20°C. Subsequently, aliquots containing 40 µg of protein were prepared in triplicate for denaturing polyacrylamide gel electrophoresis on a pre‐cast Invitrogen Bolt 4–12% Bis‐Tris Plus gel after heating for 5 min at 95°C. Protein separation, excision of gel slices (four per loaded sample), reduction and alkylation, tryptic digestion and analysis by nLC (Proxeon EASY) connected to a LTQ‐Orbitrap XL (Thermo Electron) were performed as described previously (Oosterkamp *et al.*, 2013; Buntin *et al.*, 2017). Spectra were analysed using MaxQuant 1.5.2.8 and a database of common contaminants, next to protein databases of the reported genomes of the VSL#3 strains, which were used as input (Douillard *et al.*, 2018, 16.468 sequences) and those of *Escherichia coli* K12 and BL21‐DE3 as possible contaminants. The ‘Specific Trypsin/P’ digestion mode was used in MaxQuant as described previously (Cox *et al.*, 2011) with maximally 2 missed cleavages and further default settings for the Andromeda search engine (including first search 20 ppm peptide tolerance, main search 4.5 ppm tolerance, IT‐MS‐MS fragment match tolerance of 0.5 Da, carbamidomethyl (C) set as a fixed modification, while variable modifications were set for protein *N*‐terminal acetylation and M oxidation, which were completed by non‐default settings for de‐amidation of N and Q; the maximum number of modifications per peptide was 5). The ‘label‐free quantification’ and the ‘match between runs’ options were enabled. De‐amidated peptides were allowed to be used for protein quantification, and all other quantification settings were kept default. Filtering and further bioinformatic analysis of the MaxQuant/Andromeda workflow output and the analysis of the abundances of the identified proteins were performed with the Perseus 1.5.5.3 module (Tyanova *et al.*, 2016). Accepted were peptides and proteins with a false discovery rate (FDR) of < 1% and proteins with at least two identified peptides of which at least one should be unique and at least one should be unmodified. Reversed hits were deleted from the MaxQuant result table. The normal logarithm was taken from protein LFQ MS1 intensities as obtained from MaxQuant. Zero ‘Log LFQ’ values were replaced by a value of 4.3 (a value slightly lower than the lowest measured value) to make sensible ratio calculations possible. Relative protein quantitation of the two sample lots was done with Perseus by applying a two‐sample *t* test using the ‘LFQ intensity’ columns. Protein groups that were found to deviate by more than a factor of 10 (log abundance < −1 or > 1) and a *P*‐value smaller than 0.01 (−log *P*‐value > 2) were considered to be significantly different. The nLC‐MSMS system quality was checked with PTXQC using the MaxQuant result files, as described previously (Bielow *et al.*, 2016).

## Conflict of interest

DM and WMdV are in the Scientific Advisory Board of Actial Farmaceutica SRL.

## Supporting information


**Fig. S1**. qPCR calibration curves for each VSL#3 species. *C* ^t^ values vs. Log10 FU is repeated together with the linear regression line equation and tge *R* ^2^ value. The code of the VSL#3 strains used to build the calibration curves is repeated. FU, Flouorescence Units because cells have been counted by flow cytometry.

**Table S1.** Species‐specific primers targeted to the *pyrK* gene of VSL#3 species.

**Table S2.** Differentially abundant protein species detected by metaproteomics in VSL#3 lots B and C.
