# Supplementary figures and images for: Development of omics‐based protocols for the microbiological characterization of multi‐strain formulations marketed as probiotics: the case of VSL#3
Source: Microb Biotechnol. 2019 Aug 12;12(6):1371–86. doi: 10.1111/1751-7915.13476 (PMC6801179; doi:10.1111/1751-7915.13476)

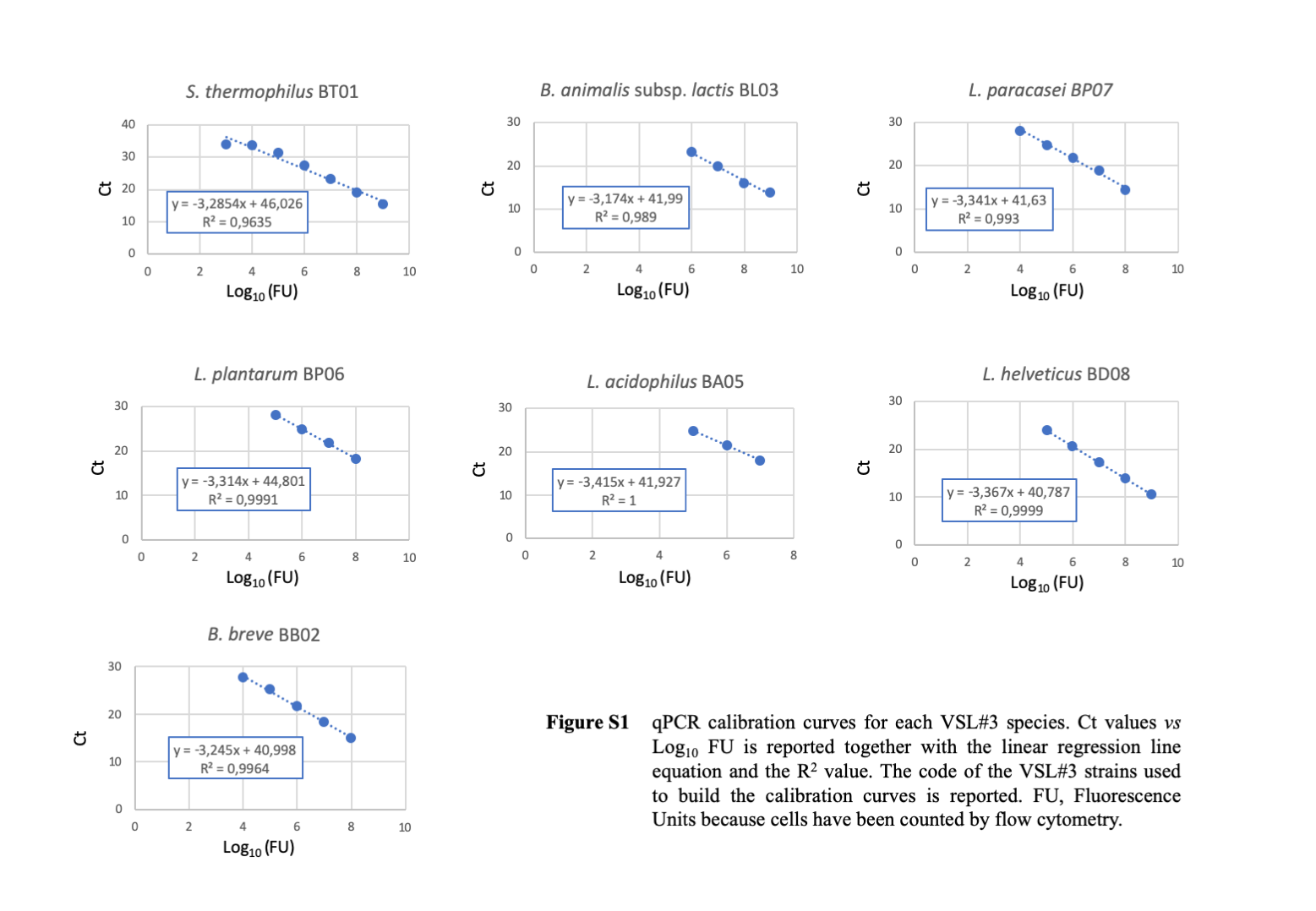

Supplement: Supplementary file 1 — Fig. S1. qPCR calibration curves for each VSL#3 species. C t values vs. Log10 FU is repeated together with the linear regression line equation and tge R 2 value. The code of the VSL#3 strains used to build the calibration curves is repeated. FU, Flouorescence Units because cells have been counted by flow cytometry. [file MBT2-12-1371-s001.tiff]
