# Supplementary material for: Development of omics‐based protocols for the microbiological characterization of multi‐strain formulations marketed as probiotics: the case of VSL#3
Source: Microb Biotechnol. 2019 Aug 12;12(6):1371–86. doi: 10.1111/1751-7915.13476 (PMC6801179; doi:10.1111/1751-7915.13476)
Supplement: Supplementary file 2 — Table S1. Species‐specific primers targeted to the pyrK gene of VSL#3 species. [file MBT2-12-1371-s002.docx]

Table S1 Species-specific primers targeted to the *pyrK* gene of VSL#3 species

| Target bacterial species | Code and primer sequence | Expected dimension (bp) of PCR fragment | |
| --- | --- | --- | --- |
| *S. thermophilus* | StF 5’-gtggttaccatgtgagaagttgaaac-3’ | | 142 |
|  | StR 5’-ttgaaatccgtggtggcaagaaatt-3’ | |  |
|  |  | |  |
| *L. acidophilus* | LaF 5’-gtccaaatgctgacatcgttgcaat-3’ | | 140 |
|  | LaR 5’-tagcaacttctgctgccttttcaaac-3’ | |  |
|  |  | |  |
| *L. helveticus* | LhF 5’-cgacattaagttcggtttacaatacggt-3’ | | 151 |
|  | LhR 5’-taccttcttgtgattcaatcttagggaa-3’ | |  |
|  |  | |  |
| *L. paracasei* | LprF 5’-gacaactcttgaagataccccgaatg-3’ | | 144 |
|  | LprR 5’-gccaacgtgagtgtcatcataaagg-3’ | |  |
|  |  | |  |
| *L. plantarum* | LpF 5’-cagccaaagtgttttcagcctttt-3’ | | 122 |
|  | LpR 5’-acgctgtctttgatggtactgac-3’ | |  |
|  |  | |  |
| *B. animalis* subsp. *lactis* | BlF 5’-gaaccggcgaatgcacca-3’ | | 129 |
|  | BlR 5’-atggctgtggaaatgccgttc-3’ | |  |
|  |  | |  |
| *B. breve* | BbF 5’-cactggctggctctgtctt-3’ | | 139 |
|  | BbR 5’-acgatcttgtcgccgtcaaca-3’ | |  |
